# The halophilic archaeon *Halogranum roseipondis* sp. nov. is susceptible to a virus carrying an exceptionally high number of viral tRNA genes

**DOI:** 10.1128/spectrum.00854-26

**Published:** 2026-08-06

**Authors:** Mirka Lampi, Erika Nordman, Pavlina Gregorova, Janne J. Ravantti, L. Peter Sarin

**Affiliations:** 1 RNAcious Laboratory, Department of Molecular and Integrative Biosciences, Faculty of Biological and Environmental Sciences, University of Helsinki 89214, Helsinki, Finland; 2 Doctoral Programme in Integrative Life Science, University of Helsinki Doctoral School, University of Helsinki 3835, Helsinki, Finland; 3 Department of Molecular and Integrative Biosciences, Faculty of Biological and Environmental Sciences, University of Helsinki 89214, Helsinki, Finland; 4 HiLIFE – Helsinki Institute of Life Science, University of Helsinki 3835, Helsinki, Finland; North Carolina State University, Raleigh, North Carolina, USA

**Keywords:** viral tRNA, haloarchaeon, archaeal virus, *Halogranum*

## Abstract

Archaea constitute a diverse group of organisms, many of which inhabit extreme environments, such as haloarchaea that dominate hypersaline ecosystems, like solar salterns. Sampling of solar salterns and other hypersaline environments has resulted in numerous haloarchaeal isolates, including 3 classified and 27 uncharacterized *Halogranum* species. However, no complete genome has so far been reported for any member of this genus. Here, we present the first comprehensive study of *Halogranum* sp. SS5-1 isolated from a solar saltern in Samut Sakhon, Thailand. *Hgn*. SS5-1 is a pleomorphic, aerobic heterotroph that thrives in high salinity and moderate temperature and is capable of hydrolyzing starch. Its genome consists of a 3.6 Mbp chromosome and seven additional plasmids. Based on our phylogenetic analyses, which establish *Hgn*. SS5-1 as a distinct species, we propose that it will be classified as the novel species *Halogranum roseipondis* sp. nov. SS5-1^T^. Additionally, we report that *Hgn. roseipondis* sp. nov. SS5-1^T^ is infected by *Hagravirus capitaneum* (HGTV-1), the only virus known to infect a *Halogranum* host. HGTV-1 exhibits a unique head-tailed morphology and encodes the largest archaeal virus double-stranded DNA genome known to date, including 34 tRNA-encoding genes. Codon usage analysis of the viral genome suggests partial alignment with host preferences, yet the abundance of viral tRNA genes hints at broader roles, potentially including roles in translation and host regulation. This study establishes *Hgn. roseipondis* and HGTV-1 as a novel virus–host system, opening avenues to explore infection dynamics and the roles of virus-encoded tRNA in archaea.

**IMPORTANCE:**

Archaea that thrive in high-salinity environments are key players in geochemical cycles and important contributors to ecosystem productivity. Despite their ecological significance and importance for the development of novel methodologies in synthetic biology, haloarchaea remain poorly studied. Further exploration of haloarchaea is required to obtain valuable information on the evolution of cellular complexity and the molecular mechanisms that allow cells to thrive in harsh environmental conditions. Here, we present the characterization of a novel archaeon, *Halogranum roseipondis* sp. SS5-1^T^, alongside the infection cycle of its associated virus, *Hagravirus capitaneum*. This tailed myovirus carries an extraordinary set of 34 viral tRNA genes, a feature that opens intriguing questions about virus–host interactions and translational control. Our findings lay the groundwork for future investigations into the expression and function of viral tRNAs in an archaeal model system, thereby opening a new frontier for studying archaeal translation and virus-driven modulation of host cellular processes.

## INTRODUCTION

Archaea constitute one of the three domains of life and include a diverse group of extremophiles that thrive in seemingly uninhabitable environments, such as acidic hot springs (1), hydrothermal vents (2), and soda lakes (3). Among these extremophiles, haloarchaea inhabit hypersaline environments, in which they are often the dominant microorganisms, outcompeting halophilic bacteria and eukaryotes (4). Such hypersaline conditions are encountered in, e.g., marine solar saltern ponds, which are man-made environments where seawater is evaporated to crystallize salt. The salinity of these shallow evaporation ponds may vary between that of typical seawater and crystallized salt. Unlike most extreme habitats, evaporation ponds are easily accessible. Hence, they have been extensively sampled and studied, uncovering archaea mainly belonging to the order *Halobacteriales* and families *Haloferacaceae* and *Haloarculaceae* (5–9). *Haloferacaceae* consists of 26 genera, of which *Haloferax* and *Halorubrum* contain 23 and 50 classified species, respectively, whereas most other genera are small and include three or fewer species (10, 11). Of these, genus *Halogranum* contains 3 characterized and 27 uncharacterized species, the latter mainly discovered following a systematic search for halophilic viruses from a solar saltern in Samut Sakhon, Thailand (5, 6). Many haloarchaeal virus isolates are reported to have a wide host range, especially because species belonging to *Halorubrum* and *Haloarcula* are susceptible to infection by several different virus isolates. However, of the 85 haloarchaeal viruses tested on *Halogranum* isolates so far, only one is known to infect a *Halogranum* host (5, 6).

To date, genus *Halogranum* contains three characterized species, all isolated from solar salterns. This genus was originally created based on *Halogranum rubrum* (12) and later expanded with *Halogranum salarium* (13), *Halogranum amylolyticum*, and *Halogranum gelatinilyticum* (14), of which *Hgn. salarium* has recently been phylogenetically reclassified as a strain of *Hgn. rubrum* (15). All characterized *Halogranum* species are extremely halophilic, thriving at a total salinity range of 2.6–3.9 M (12–14). In this study, we present the first detailed characterization of the haloarchaeon *Halogranum* sp. SS5-1 (5). We show that *Hgn*. sp. SS5-1 cells are pleomorphic, non-motile, aerobic heterotrophs with optimal growth at 42°C, pH 6–7.5, in the presence of 3.4 M salt. *Hgn*. sp. SS5-1 utilizes a variety of single-carbon sources, and it hydrolyzes starch but not gelatin. Furthermore, we also present the complete genome of *Hgn*. sp. SS5-1—the first complete genome in the *Halogranum* genus—consisting of a 3,617 kbp double-stranded DNA (dsDNA) main chromosome and seven additional plasmids that range in size from 40 to 561 kbp. Using Roary pangenomic analysis, we group *Hgn*. sp. SS5-1 to the *Halogranum* family next to the *Haloprofundus* genus. Similarity analysis of the three 16S ribosomal RNA genes and the average nucleotide identity (ANI) analysis further suggests that *Hgn*. sp. SS5-1 constitutes a distinct novel species that is closely related to *Hgn. rubrum* and *Hgn. amylolyticum*. Consequently, we propose that *Hgn*. sp. SS5-1 should be classified as a novel species within the *Halogranum* genus, bearing the designation *Halogranum roseipondis nov*. SS5-1^T^, wherein *rosei* refers to the pink color of the species in liquid culture, and *pondis* refers to the isolation pond, i.e., solar saltern.

In addition, we also provide the first infection cycle characterization for a virus infecting an archaeon from genus *Halogranum: Hagravirus capitaneum* (previously known as Halogranum tailed virus 1 [HGTV-1]) (5, 16). *Hagravirus capitaneum* belongs to the order *Thumleimavirales*, representing the only known species in the family *Halomagnusviridae* and the genus *Hagravirus*. As is typical for infective head-tailed viruses known to date, HGTV-1 replicates using a lytic cycle with progeny virions being released into the cell’s surroundings. We show that the HGTV-1 infection cycle takes approximately 10 h, terminating with a modest amount of progeny virus production. We also demonstrate that HGTV-1 has an unusual morphotype with an icosahedral capsid, connected to the tail by a long and thin neck structure. Interestingly, HGTV-1 has the largest dsDNA genome of any known archaeal virus to date. Reanalysis of tRNA genes established that it carries 34 virus-encoded tRNA (vtRNA) genes covering all amino acids in the universal genetic code, except for histidine. Previous studies have suggested that vtRNAs may bridge biases caused by codon usage differences in the host and virus genomes (17). Our codon usage comparison revealed slight biases that might explain the presence of some of the vtRNA genes in the HGTV-1 genome but not all of them. Hence, we hypothesize that in addition to bridging codon usage differences, vtRNAs might partake in other infection-supporting roles, such as acting as translational decoys (18), for which additional studies are needed. Importantly, this constitutes the first detailed host–virus characterization of a *Halogranum* infection model, paving the way for further studies on viral tRNA functions in an archaeal host organism.

## RESULTS

The *Halogranum* isolate investigated in this study has previously been published as *Halogranum* sp. SS5-1 and is now proposed as a new species with the name *Halogranum roseipondis* SS5-1^T^ (see Protolog). It is hereafter referred to as *Hgn. roseipondis*.

### *Halogranum roseipondis* is an extreme halophile able to utilize several single-carbon sources

*Halogranum roseipondis* SS5-1^T^ is a non-motile, obligate aerobe that was originally isolated from a salt crystallization pond in Samut Sakhon, Thailand (Table 1; see Fig. S1 at https://doi.org/10.5281/zenodo.21291954) (5). *Hgn. roseipondis* SS5-1^T^ forms round (~2 mm in diameter) and shiny colonies with a distinct pink coloration, probably due to carotenoid production, a common trait in many haloarchaea (19). Pigment production diminishes as growth temperatures exceed 45°C, with colonies acquiring a yellowish/light orange color (see Fig. S2 at https://doi.org/10.5281/zenodo.21291954). We determined that *Hgn. roseipondis* grows at a temperature range from 25°C to 45°C with an optimum at 42°C when assayed in liquid microcultures (Table 1; see Fig. S3 at https://doi.org/10.5281/zenodo.21291954). However, growth can be sustained at 50°C both on solid media and in liquid flask cultures, although aggregates frequently appear at >45°C, suggesting that the cells are at their survivability limit. Next, we characterized the impact of salinity at different temperatures. *Hgn. roseipondis* grows optimally in 23% modified growth medium (MGM) (3.4 M total salts) at 42°C (Table 1; see Fig. S3 at https://doi.org/10.5281/zenodo.21291954), whereas a ~2 M (12% MGM) minimal salt concentration is required for growth. *Hgn. roseipondis* cells remain intact in 10% (wt/vol) NaCl (1.71 M) solutions, but lysis is immediate in ddH_2_O. Conversely, growth was still detected at 28% MGM (4.1 M total salts), although liquid cultures are difficult to handle at such high salinity due to salt crystallization. Interestingly, we also observed that 12%–18% MGM (1.8–2.6 M total salinity) concentrations were more favorable at lower (<37°C) growth temperatures (see Fig. S3 at https://doi.org/10.5281/zenodo.21291954). We also established that *Hgn. roseipondis* has a narrow pH range of 6.0–7.5 with a preference for neutral toward slightly acidic environments (Table 1; see Fig. S4 at https://doi.org/10.5281/zenodo.21291954). Furthermore, *Hgn. roseipondis* can grow on several single-carbon sources, and it hydrolyzes starch but not gelatin (Table 1; see Fig. S5 at https://doi.org/10.5281/zenodo.21291954). We also concluded that growth is inhibited by novobiocin, mevinolin, and rifampicin, whereas no inhibition was observed with other antibiotics tested (Table 1; see Fig. S6 at https://doi.org/10.5281/zenodo.21291954). Hence, we conclude that optimum laboratory growth conditions for *Hgn. roseipondis* are achieved on 23% MGM media at 42°C and pH 6.5–7.

**TABLE 1:** Characteristics of *Hgn. roseipondis* SS5-1^T^ and reference strains*^a^*

	*Halogranum roseipondis* SS5-1^T^	*Halogranum salarium* B-1	*Haloarcula vallismortis*	*Haloferax gibbonsii* LR2-5
Salinity range for growth in MGM (M)	2.7–4.2	2.7–4.2	2.7–4.2	2.7–4.2
Minimal salt concentration to prevent cell lysis (M)	1.7	0.51^*b*^	n.d.	n.d.
pH optimum	6.5–7.0	6.0–6.5	7.5^*c*^	7.0^*d*^
Temperature range (optimum in 23% MGM)	25°C–50°C (42°C)	25°C–50°C (42°C)	30°C–50°C (42°C)	20°C–50°C (42°C)
Anaerobic growth	–	–	–	–
Cell size (µm)	1–2 (irregular)	1–3 (irregular)	2–5^*c*^ (irregular)	1.5–4 (irregular)
Motility, microscopy	–	+	+^*c*^	+^*d*^
Motility, plate	–	–	–	+
Catalase	+	+	+	+
Oxidase	+	+	+	+
Gelatin hydrolyzation	–	+	–	+
Starch hydrolyzation	+	+	–	–
Antibiotic resistance				
Ampicillin	+	+	+	+
Tetracycline	+	+	+	+
Novobiocin	–	–	–	–
Mevinolin	–	–	–	–
Rifampicin	–	–	–	–
Carbon source utilization				
Fructose	++	+	++	++
Glucose	++	+	++	++
Galactose	–	+	+	–
Glycerol	++	++	++	+
Lactose	–	–	–	+
Mannitol	–	+	+	++
Sucrose	++	+	++	++
Na-succinate	+	–	–	–
Na-lactate	++	++	–	++
Na-acetate	+	+	–	–

^*a*^ +, positive, grows; ++, grows well; −, negative, no growth; n.d., not determined.

^*b*^ (20).

^*c*^ (21, 22).

^*d*^ (23).

### *Hgn. roseipondis* cells are irregularly shaped and feature club-like surface structures

**Fig 1:**
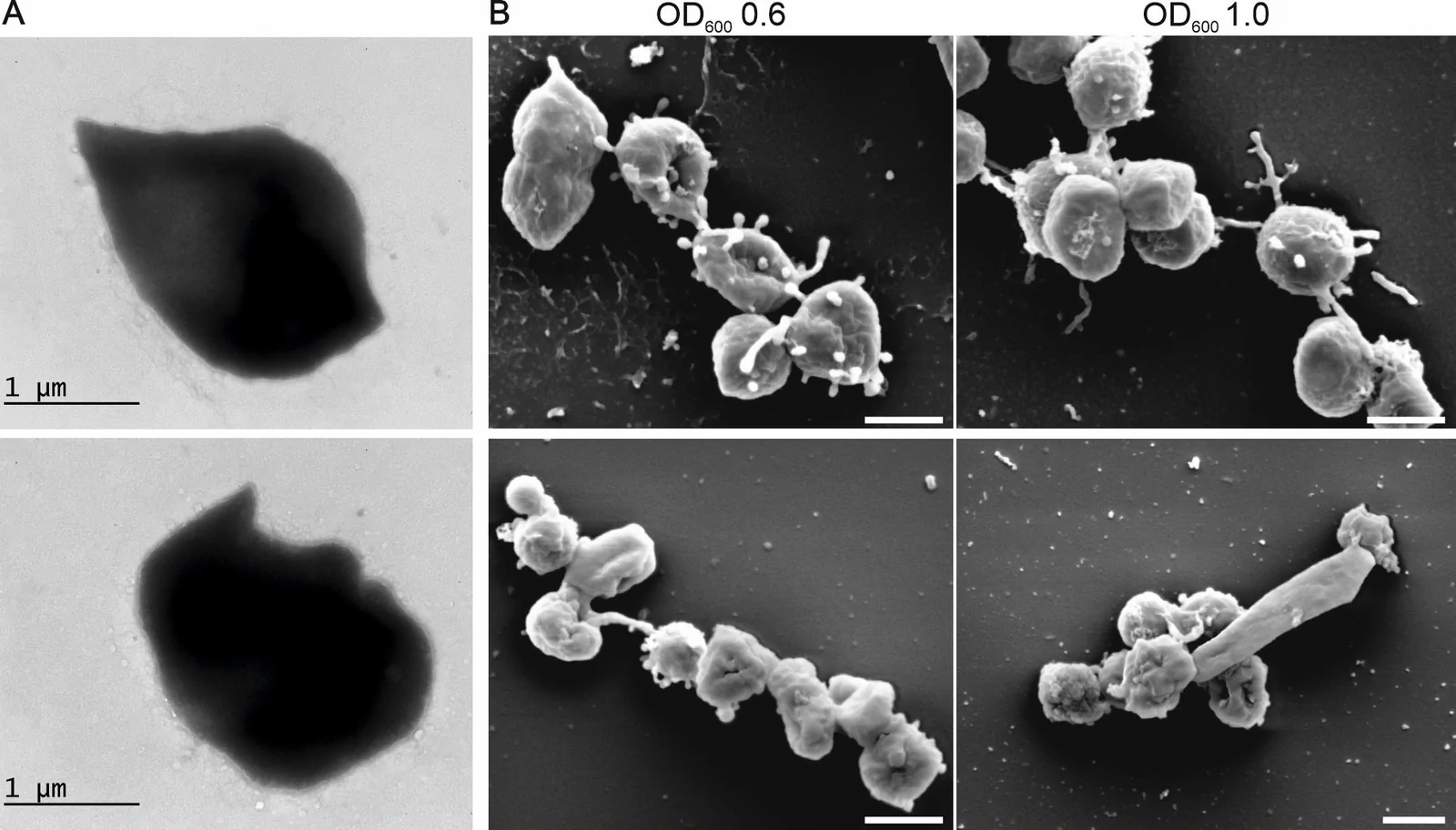
*Halogranum roseipondis* SS5-1 cells are irregularly shaped. (**A**) Negative-stain (2% uranyl acetate) transmission electron microscopy of *Hgn. roseipondis* grown to OD_600_ of 1.0. (**B**) Scanning electron micrographs of *Hgn. roseipondis* grown to OD_600_ of 0.6 or 1 (see Materials and Methods). The average cell diameter is 1–1.5 µm. The scale bars in panel B correspond to 1 µm.

Our initial light microscopy and transmission electron microscopy (TEM) findings suggested that *Hgn. roseipondis* might have an irregular cell morphology (see Fig. S7 at https://doi.org/10.5281/zenodo.21291954; Fig. 1A). Similar observations have been made for other haloarchaea, such as *Haloferax gibbonsii*, where cell morphology changes are tightly linked to the growth phase (23). Consequently, we set out to investigate if such a connection is present in *Hgn. roseipondis* by examining its cellular morphology at two growth phases. In the early exponential growth phase (optical density at 600 nm [OD_600_] 0.6), cells are irregularly shaped, although we noticed a slight prevalence toward coccoidal cells with a diameter of ~1 µm (Fig. 1B), whereas cells at a later exponential growth phase (OD_600_ 1) appeared more irregular, elongated, and angular with varying sizes being observed (Fig. 1B). Irrespective of growth phase, some cells feature club-like structures or surface protrusions, which seem to connect some of the cells together (Fig. 1B). It has previously been reported that archaea express unique attachment and anchoring structures, like hami (24), cannulae (25, 26), and pili, which facilitate adhesion and biofilm formation (27), as well as archaeal flagella, i.e., archaella, for motility (28) and intriguing intercellular bridges that possibly facilitate the exchange of cellular components (29). However, the *Hgn. roseipondis* genome does not carry genes for archaeal flagella. Furthermore, these club-like surface structures do not directly resemble any previously reported structures, and their function remains to be determined.

### Phylogenetic analyses suggest that *Hgn. roseipondis* constitutes a novel *Halogranum* species

**Fig 2:**
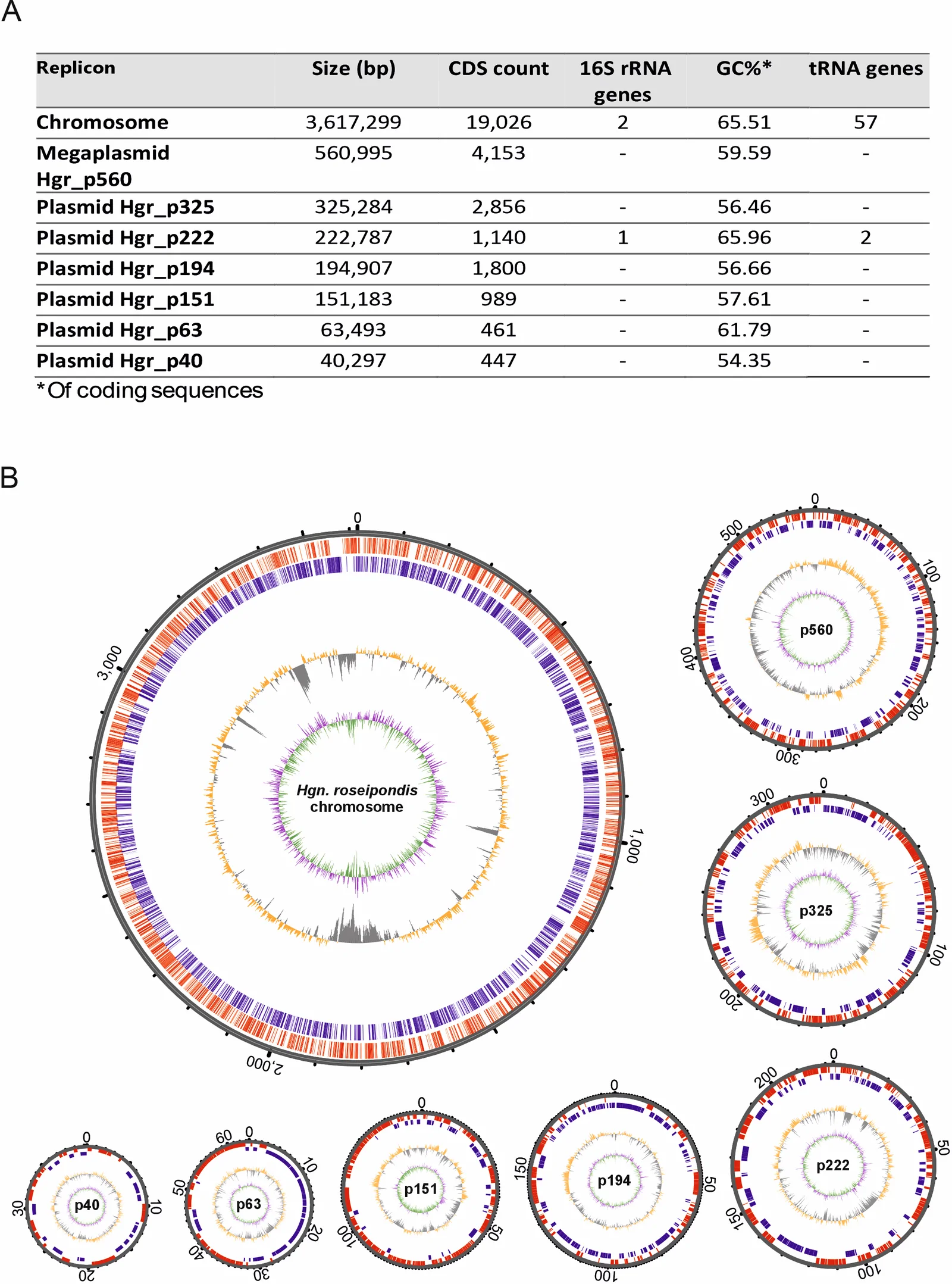
The *Halogranum roseipondis* SS5-1^T^ genome consists of a large chromosome and seven additional DNA replicons. (**A**) Key numerical details of the eight *Hgn. roseipondis* genome replicons. (**B**) Circular genome maps of each genome molecule. From outside to center: coding DNA sequence (CDS) forward strand and CDS reverse strand presented in red and blue, respectively. The middle circle (yellow/gray) represents GC content distribution, and the innermost circle (green/purple) shows the GC skew. Size annotations on the outer ring of the map are in kilobases. To facilitate legibility, circular map sizes are not to scale.

The *Hgn. roseipondis* genome was sequenced using a PacBio Sequel II sequencer and assembled using Pacbio’s Microbial Genome Analysis application. A total of 10,761 PacBio HiFi sequence reads were obtained, with an N50 read length of 16,828 bp (381–43,667 bp), resulting in an average coverage depth of 18.7–44.2× (see Table S1 at https://doi.org/10.5281/zenodo.21291954). The *Halogranum roseipondis* genome consists of a large 3,617 kbp chromosome, a 560 kbp megaplasmid, and six additional plasmids ranging between 40 and 325 kbp in size (Fig. 2). The chromosomal GC content is 65.51%, and plasmid GC contents differ to some extent, varying between 54.35% and 65.96%.

**Fig 3:**
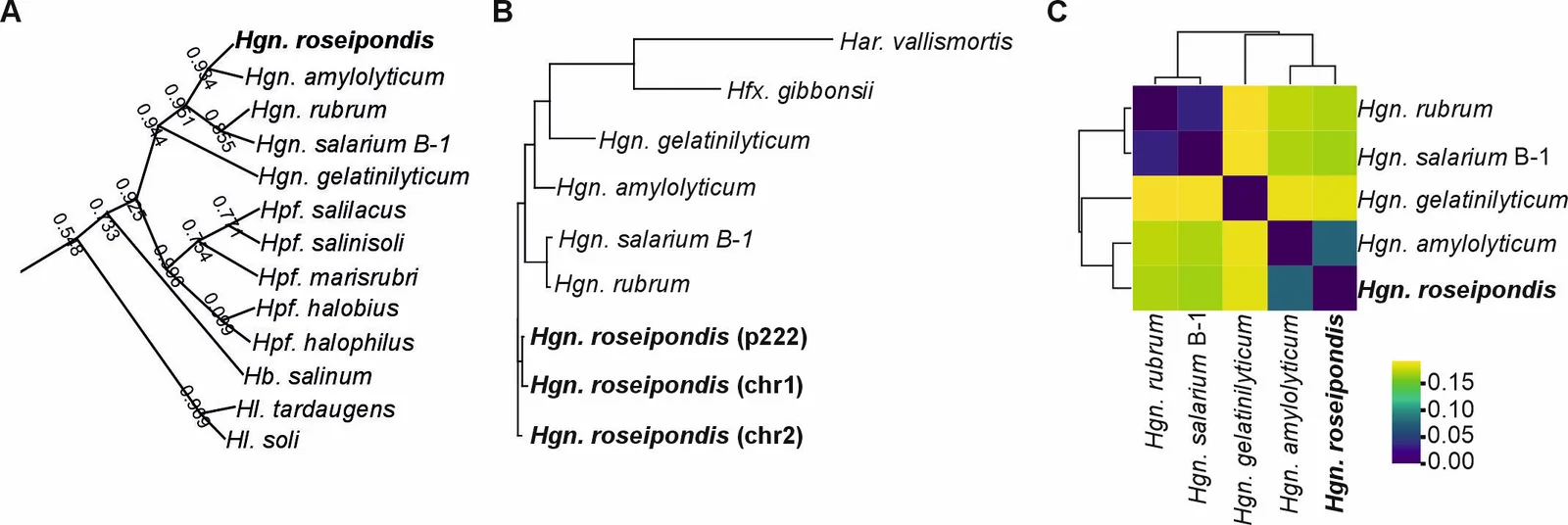
Phylogenetic relationship of *Hgn. roseipondis* SS5-1^T^. (**A**) Roary pangenomic analysis places *Hgn. roseipondis* on the same branch as *Hgn. amylolyticum*. Genera abbreviations: *Hl*., *Halegenticoccus*; *Hb*., *Halobium*; *Hpf*., *Haloprofundus*. Branch lengths represent gene content dissimilarity based on pangenome presence/absence matrices. For the complete tree, including 125 *Haloferacaceae* species, see Fig. S7 (https://doi.org/10.5281/zenodo.21291954). (**B**) Phylogenetic tree of 16S rRNA gene similarity within the genus *Halogranum* and the reference strains used in this study. The locations of *Hgn. roseipondis* 16S rRNA genes are presented in brackets. Chr1, in chromosome, location 1,345,500–1,346,971; Chr2, in chromosome, location 2,929,224–2,930,695; p222, in plasmid Hgr_p222. (**C**) OrthoANI analysis of *Halogranum* species. The color legend represents OrthoANI value fractions, where a higher value indicates less similarity. For the complete ANI analysis with 125 *Haloferacaceae* species, see Fig. S9 (https://doi.org/10.5281/zenodo.21291954).

Next, we determined how the *Hgn. roseipondis* genome relates to other haloarchaea. Roary pangenomic analysis (30–32) places *Hgn. roseipondis* in the same cluster as other *Halogranum* species when compared to representative type species from all genera within the family *Haloferacaceae*, except for *Candidatus Haloextosymbiotes* (Fig. 3A; see Table S2; Fig. S8 at https://doi.org/10.5281/zenodo.21291954). This is supported by phylogeny based on 16S rRNA sequence similarity, which places *Hgn. roseipondis* next to *Hgn. rubrum* and *Hgn. amylolyticum* (Fig. 3B). Notably, two 16S rRNA genes are present in the *Hgn. roseipondis* chromosome and one in the plasmid Hgr_p222 (Fig. 2A). There is a minor intragenomic variance between the two chromosomal 16S rRNA genes (99.7% sequence similarity). Likewise, 99.7% and 99.5% sequence similarities are observed when comparing the plasmid-based 16S rRNA gene to the respective chromosomal genes. Furthermore, ANI analysis suggests that *Hgn. roseipondis* should be classified as a novel *Halogranum* species, as the highest ANI score obtained is 92.28 (to *Hgn. amylolyticum*), which exceeds the 5% threshold and is sufficiently different for *Hgn. roseipondis* to be considered as a novel species (Fig. 3C; see Fig. S9 at https://doi.org/10.5281/zenodo.21291954) (33, 34).

### Protolog

*Halogranum* species, *Halogranum roseipondis* SS5-1^T^ sp. nov. *Roseipondis* (ro.se.i.pon′dis. L. adj. roseus, rosy; L. n. pondus, pond; N.L. gen. n. roseipondis, of a rosy pond), referring to the pink coloration of the liquid culture and the hypersaline pond from which this species was isolated.

*Halogranum roseipondis* SS5-1^T^ is a pleomorphic haloarchaeon that grows at near-neutral conditions (pH 6.5–7) in a wide range of salinities (2.7–4.2 M) and temperatures (25°C–50°C). *Hgn. roseipondis* SS5-1^T^ has an aerobic metabolism. It utilizes a variety of carbon sources, including fructose, glucose, glycerol, sucrose, Na-succinate, Na-lactate, and Na-acetate, and it hydrolyzes starch but not gelatin. It is positive for catalase and oxidase activity (Table 1) and is sensitive to rifampicin, mevinolin, and novobiocin (Table 1). *Hgn. roseipondis* SS5-1^T^ produces a red/pink pigment and may feature extracellular surface appendages that seemingly connect the cells (Fig. 1). *Hgn. roseipondis* SS5-1^T^ can be infected with *Hagravirus capitaneum* HGTV-1.

*Halogranum roseipondis* SS5-1^T^ belongs to the genus *Halogranum* within the family *Halobacteriaceae*, class *Halobacteria*, domain *Archaea*. Phylogenetic analysis based on 16S rRNA gene sequences places *Hgn. roseipondis* next to *Halogranum amylolyticum* and *Halogranum rubrum*. Average nucleotide identity by orthology (OrthoANI) analysis reveals the ANI value to the closest relative, *Halogranum amylolyticum*, to be 92.28. The *Hgn. roseipondis* SS5-1^T^ genome consists of a 3,617,299 bp main chromosome (GC% 65.51) and seven additional plasmids ranging from 40,297 to 560,995 bp in size (Fig. 2).

### *Hgn. roseipondis* is infected by a virus with an unusual head-tailed morphotype

**Fig 4:**
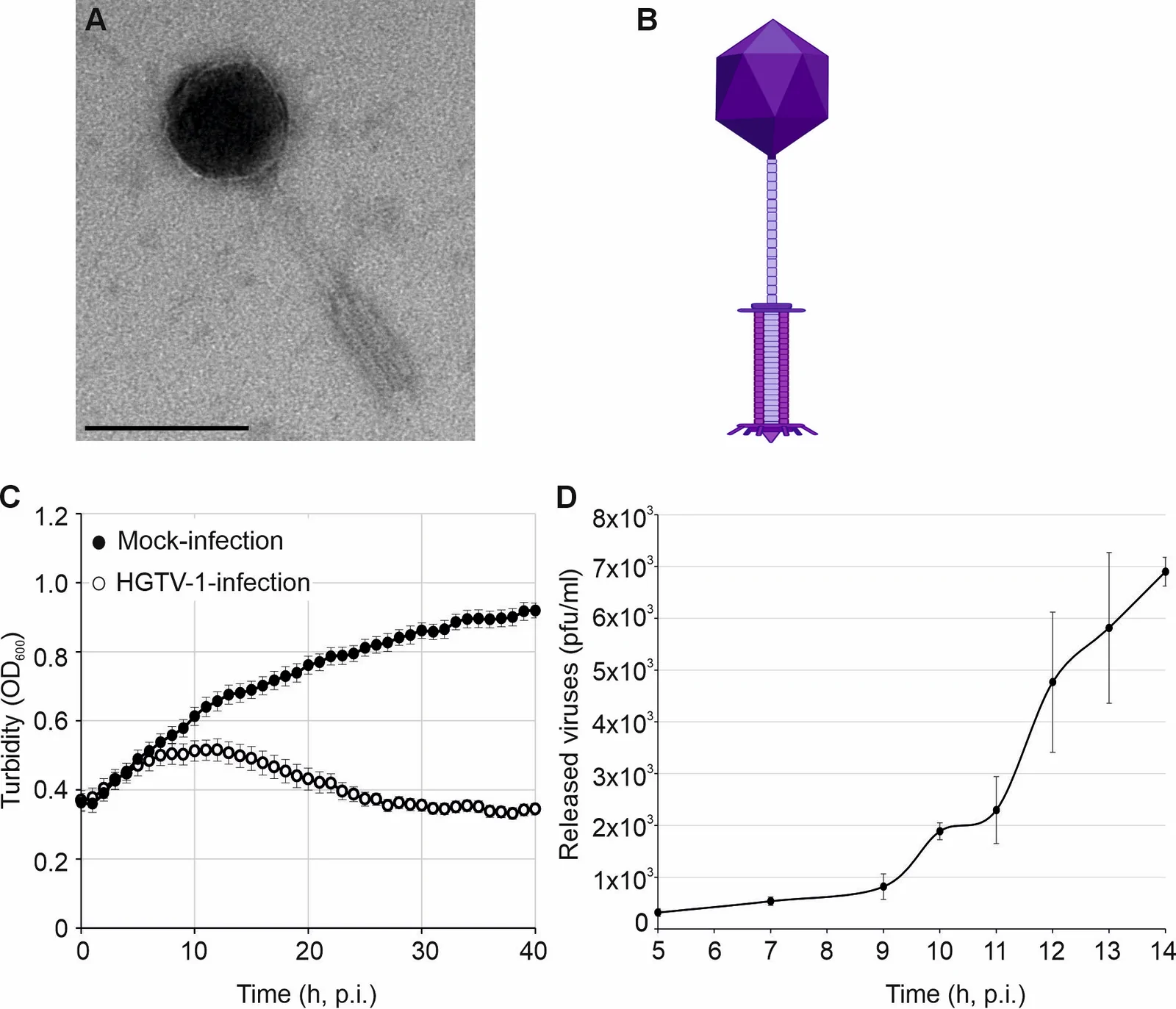
HGTV-1 has an unusual head-tailed morphotype and lytic infection cycle. (**A**) A transmission electron micrograph of the HGTV-1 virion shows a thin, long neck that connects the tail to the capsid. (**B**) Schematic interpretation of the HGTV-1 virion (based on panel A). (**C**) Representative growth curves of HGTV-1 infected and mock-infected *Hgn. roseipondis* cultures. Note that the turbidity of the infected culture begins to decrease at 12 h p.i. (**D**) One-step growth curve of HGTV-1 infected *Hgn. roseipondis* cells. Release of progeny virions is observed at 10 h p.i., followed by increasing numbers of released virions. Error bars denote standard deviation (*n* = 3).

Many archaeal viruses are known for their extraordinary virion morphologies (35). The HGTV-1 virion has a total length of ~230 nm and represents a rather “classical” head-tailed morphotype with an icosahedral capsid (~75 nm in diameter) and a long tail (~155 nm) (Fig. 4A and B). Nevertheless, the tail structure is exceptional, as it is composed of a thin neck and a much wider clamp-like expansion (Fig. 4A and B). Moreover, based on frequent electron microscopy observations, the tail structure seems to easily detach from the virion (see Fig. S9 at https://doi.org/10.5281/zenodo.21291954), although it is not clear whether this is solely an artifact of sample preparation. We also identified tail assemblies of various lengths, some 3–4× the length of the mature tail structure, which might be non-processed assembly intermediates (see Fig. S10 at https://doi.org/10.5281/zenodo.21291954). However, neither virion assembly nor the function of the clamp-like expansion is within the scope of this work, and further studies are needed to address these aspects.

It has previously been reported by Atanasova et al. that HGTV-1 infects *Haloarcula vallismortis*, albeit with very low infectivity (5). The authors also performed a broad host range screening of HGTV-1 against 32 archaeal species, which did not reveal any additional susceptible hosts (5). Our cross-infection studies confirmed these results, in addition to which we did not observe any infectivity with the reference strains *Hgn. salarium* or *Hfx. gibbonsii* (see Fig. S11 at https://doi.org/10.5281/zenodo.21291954).

HGTV-1 infects *Hgn. roseipondis* most efficiently at 37°C using 23% MGM growth medium (Fig. 4). At higher temperatures and/or salinities, the host becomes more resistant to infection, possibly since the conditions are less favorable to virus adsorption, allowing the host culture to outgrow the infection (see Fig. S12 at https://doi.org/10.5281/zenodo.21291954). The HGTV-1 infection cycle is lytic, and infected cells burst at ~12 h post-infection (p.i.), as is noticeable from the declining turbidity of the host culture (Fig. 4C). However, the infection cycle is not fully synchronized, and nascent virions can be detected as early as 10 h p.i., followed by a subsequent increase in the number of released virions. Based on one-step growth curves, we estimate the burst size after 14 h p.i. to be modest, releasing on average only 20 virions per infected cell (Fig. 4D).

### HGTV-1 encodes numerous viral tRNAs of unknown function

HGTV-1 has previously been reported to have a genome size of 143,855 bp (16)—the largest genome of any archaeal virus known to date—and to carry 36 vtRNA genes, including two pseudogenes, constituting the highest number of vtRNA genes encoded by an archaeal virus (16). Following a reanalysis of the published genome with tRNAScan-SE 2.0 (36), we identified 34 tRNA genes—3 putative pseudogenes, 2 tRNA genes of undetermined type, and 29 regular tRNA genes with an anticodon that matches the body of the isoacceptor (see Table S3 at https://doi.org/10.5281/zenodo.21291954). Moreover, one tRNA gene also carries an intron. We also noted that the average GC content of HGTV-1 vtRNAs is somewhat lower (55.2%, range 38.6%–63.9%) than that of the host tRNAs (61%, range 50.7%–68.2%) (see Table S3 at https://doi.org/10.5281/zenodo.21291954).

**Fig 5:**
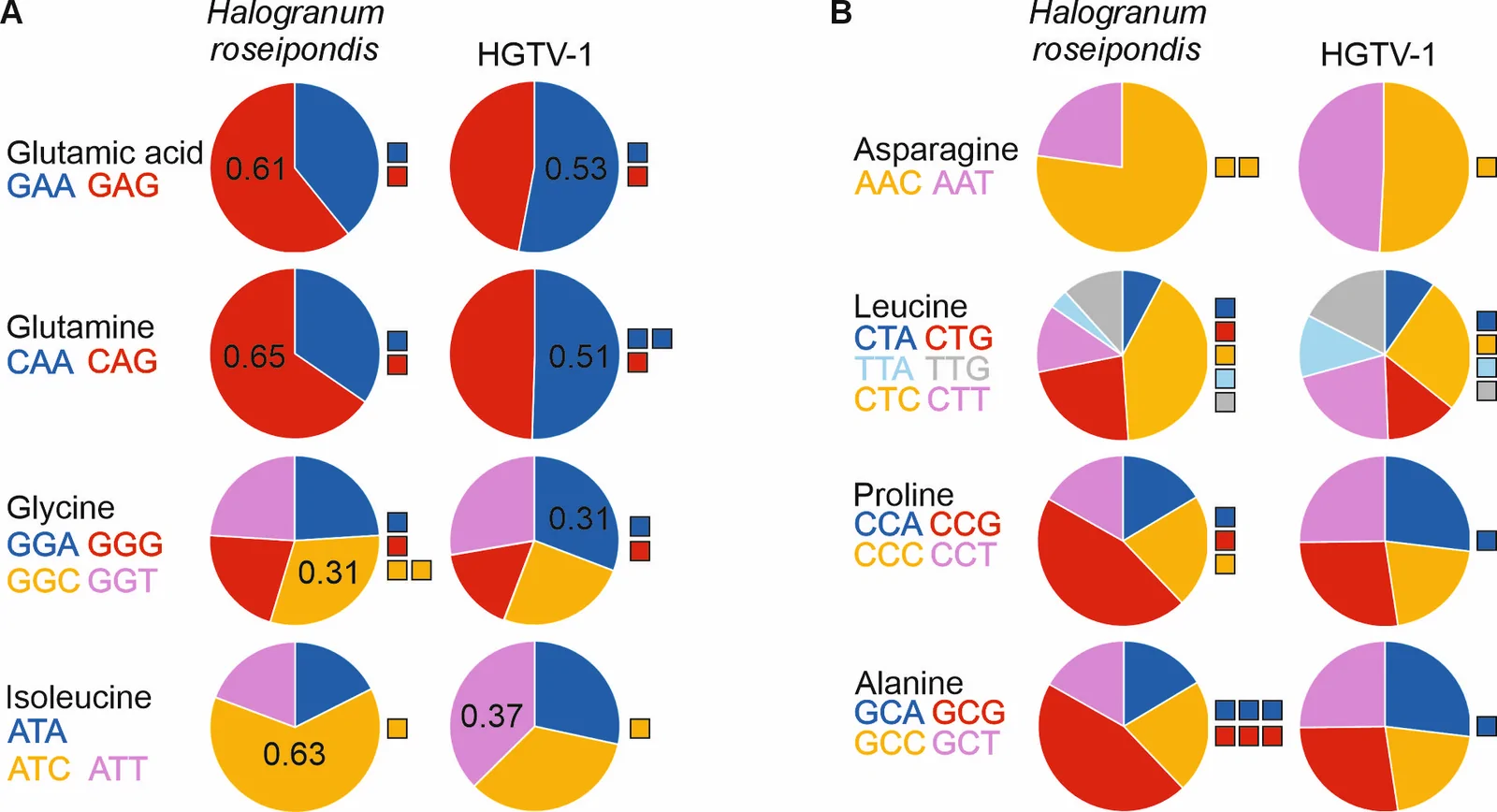
Codon usage analysis of *Hgn. roseipondis* SS5-1^T^ and HGTV-1 coding regions. (**A**) Codon usage comparison in cases where the preferred codon was different in *Hgn. roseipondis* and HGTV-1. The number in each pie chart indicates the fraction of the preferred codon. (**B**) Codon usage comparison of selected amino acids where the preferred codon is the same, but optional codons differ. Pie chart colors correspond to the color of the codons at the left side of each chart. For panels **A** and **B**, the colored squares next to the charts represent codon-matching tRNA genes carried by *Hgn. roseipondis* or HGTV-1, respectively. Each square depicts one tRNA-coding gene.

The presence of vtRNA genes is often considered a key adaptation mechanism for bridging potential discrepancies in codon usage and availability between the virus and the host (37). To investigate this further, we performed a codon usage analysis for all coding sequences in the *Hgn. roseipondis* and HGTV-1 genomes, respectively. As expected, the host encodes for tRNAs that match the preferred codons, such as GAC for aspartic acid, TTC for phenylalanine, AAC for asparagine, and TAC for tyrosine. When codon usage is non-preferential between the possible codon options, as is the case for glycine, arginine, and serine, *Hgn. roseipondis* also encodes tRNAs that match the optional codons (see Tables S3 and S4 at https://doi.org/10.5281/zenodo.21291954). However, our codon usage comparison between the host and the virus revealed differences in the preferred codons used for glutamic acid, glycine, isoleucine, and glutamine: *Hgn*. *roseipondis* favors pyrimidine (C/G) as the third codon base, whereas purine (A/T) is prevalent at the same position in HGTV-1 (Fig. 5; see Table S4 at https://doi.org/10.5281/zenodo.21291954). Importantly, these limitations can be overcome for glutamic acid, glycine, and glutamine, as HGTV-1 encodes vtRNAs with both pyrimidine and purine at the third base, thus supplementing the translation machinery with tRNAs that match transcripts originating from both genomes. Conversely, this is not the case for isoleucine, where the vtRNA matches the preferred host codon but not that of the virus (Fig. 5A). For asparagine, codon AAC is clearly more dominant in the host, whereas codons AAC and AAT are used almost equally in the virus genome. Bearing this in mind, it is surprising that HGTV-1 encodes a vtRNA for ACC only but none for AAT (Fig. 5B). In the case of leucine, HGTV-1 encodes vtRNAs for codons CTC, CTA, TTA, and TTG. CTC is the most prevalent codon in both the host and the virus, whereas optional codons CTA, TTA, and TTG are used more pronouncedly by the virus, suggesting that expression of these vtRNAs would further HGTV-1 protein translation (Fig. 5B). A similar situation is observed for proline and alanine, where the preferred codon is the same for *Hgn. roseipondis* and HGTV-1, but utilization of the A-ending codon with matching vtRNA is more prominent in the virus for optional codons (Fig. 5B). Whether viral tRNAs are recognized by the ribosome—given that their sequences differ to some extent from host tRNAs (see Fig. S13 at https://doi.org/10.5281/zenodo.21291954)—remains an open question for future studies to address.

## DISCUSSION

In this study, we provide the first detailed characterization of the physiological and genomic features of *Halogranum* sp. SS5-1 (5), based upon which we propose that it be classified as the novel species *Halogranum roseipondis* sp. nov. SS5-1^T^. Previously characterized *Halogranum* species have all been isolated from solar salterns, and they are known to grow in 2.6–3.9 M NaCl and near-neutral pH conditions (12, 20). The novel species *Hgn. roseipondis* sp. nov. SS5-1^T^ shares these fundamental growth requirements (Table 1) that align with the environmental conditions in solar salterns, where salinity and temperature may fluctuate significantly. Moreover, many haloarchaeal species produce carotenoid pigments that range in color from yellow to red (19). These carotenoids act as photoprotectors and antioxidants, protecting the cell from UV radiation damage and oxidative stress, in addition to which they have also been associated with DNA repair and membrane fluidity control (19). *Hgn. roseipondis* produces a pink/reddish pigment at optimal growth conditions (see Fig. S2 at https://doi.org/10.5281/zenodo.21291954). However, we did observe loss of pigment production at growth temperatures exceeding 45°C (see Fig. S2 at https://doi.org/10.5281/zenodo.21291954), which may be potentially attributed to transcriptional reprogramming, e.g., induction of heat shock responses, as the cells reach the limit of their survival.

Many archaea are known to feature unusual cell morphologies (38), as well as undergo growth phase-dependent morphological changes (23, 39). Our microscopic analyses revealed that *Hgn. roseipondis* cells are pleomorphic, with a predominantly coccoid morphology at the early exponential growth phase, whereas cell sizes and morphologies diversify when the culture is grown to a later exponential growth phase (Fig. 1). Furthermore, we also observed that *Hgn. roseipondis* cells are occasionally decorated with extracellular appendages, which seemingly link individual cells together (Fig. 1). These structures do not resemble any previously reported archaea-specific extracellular structures like cannulae or hami (24–26), nor have such structures been reported for any other *Halogranum* species. Although elucidating the function of these surface structures falls outside the scope of this study, it is conceivable that they may constitute a previously undescribed surface protrusion of unknown function.

The genome of *Halogranum roseipondis* represents the first complete and fully assembled *Halogranum* genome (Fig. 2). It consists of a large main chromosome, a megaplasmid, and six smaller plasmids (Fig. 2). Plasmids are common in archaea, where they often carry genes that are critical for adaptation and survival. Haloarchaea typically harbor two to seven plasmids, in addition to which they may have unique extrachromosomal elements, such as megaplasmids or minichromosomes, that are not found in other archaeal species (40). Notably, the 561 kbp megaplasmid of the *Hgn. roseipondis* genome (Fig. 2) has a significantly lower GC content than the main chromosome, a feature that is typical for haloarchaea and likewise indicative of the megaplasmid deriving from a different origin (40). Conversely, plasmid Hgr_p222 carries both rRNA and tRNA genes and has a GC content comparable to that of the main chromosome. This suggests that despite its relatively small size, Hgr_p222 could be seen as a mini-chromosome, similar to a bacterial chromid, rather than a plasmid (41, 42). The plasmids of *Halogranum roseipondis* may point to a mosaic evolutionary history, potentially enabling the integration of features such as extracellular appendages that are of uncertain origins and have not been documented in haloarchaeal species studied to date. Our phylogenetic analyses revealed that *Hgn. roseipondis* constitutes a novel *Halogranum* species, with *Hgn. amylolyticum* and *Hgn. rubrum* being its closest relatives (Fig. 3). The phenotypic properties of *Hgn. roseipondis*, *Hgn. salarium* (strain of *Hgn. rubrum* used as a reference strain in this study), and *Hgn. amylolyticum* are relatively similar, yet differences in carbon source utilization, gelatin hydrolyzation, and genome GC content were observed (Table 1; Fig. 2A) (12, 14).

Alongside the *Hgn. roseipondis* description, we report here the first characterization of HGTV-1, the only known virus infecting a *Halogranum* species. HGTV-1 displays a unique head-tailed morphology with a long, thin neck and wider tail expansion that resembles the classical contractile myovirus tail (Fig. 4). Its lytic infection cycle is relatively slow and modest in burst size (Fig. 4). This might suggest one of two potential scenarios: either it is a finely tuned host–virus interaction possibly shaped by environmental constraints or HGTV-1 infection proceeds suboptimally in *Hgn. roseipondis* since it is not the preferred host. Although the latter alternative cannot be fully excluded, neither the original cross-infection study with 32 haloarchaea (5) nor our subsequent work (see Fig. S11 at https://doi.org/10.5281/zenodo.21291954) revealed any additional hosts that HGTV-1 efficiently infected. Indeed, not even *Hgn. salarium*—the closest relative of *Hgn. roseipondis*—was susceptible to infection with HGTV-1. Such a narrow host range, combined with a slow, low-yield lytic cycle, may reflect an ecological strategy that stabilizes host populations while allowing persistent viral propagation under environmental constraints.

A particularly interesting feature of HGTV-1 is its exceptionally large number of viral tRNA genes, covering all amino acids except histidine (see Table S3 at https://doi.org/10.5281/zenodo.21291954). The function of virus-encoded tRNAs has not yet been systematically investigated in archaeal virus–host systems. However, studies in bacterial models have uncovered two main functions: viral tRNAs sustain efficient translation when host tRNA pools are depleted during infection, and they bridge codon usage discrepancies between the virus and the host by expanding the host tRNA pool (18). Our codon usage analysis suggests that most HGTV-1-encoded viral tRNAs may help bridge host–virus codon discrepancies and facilitate the translation of specific viral proteins under host-imposed constraints (Fig. 5). However, not all the HGTV-1 tRNAs fit this codon usage hypothesis, possibly implying either a more active role in the translation of host components or other alternative functions. For example, viral tRNAs may be differently modified, which could cause ribosomes translating viral transcripts to read through stop codons and/or undergo frameshifting. Some viruses are also known to induce fragmentation of tRNAs (43, 44), yielding specific tRNA fragments that have been reported to suppress antiviral defense mechanisms (45, 46) or directly inhibit translation by interfering with the host translation machinery. Although infection-induced tRNA fragments in archaea have not yet been investigated, stress-induced valine tRNA fragments in *Haloferax volcanii* are known to target the small ribosomal subunit and regulate protein synthesis (47, 48). The continuous evolutionary arms race between hosts and viruses raises the intriguing possibility that viruses may have also adapted to exploit this cellular mechanism. In conclusion, this study not only provides a detailed characterization of a novel virus–host model system but also establishes a platform for further exploration of the multifaceted mechanisms by which viruses interact with the host translation machinery.

## MATERIALS AND METHODS

### Archaeal strains and virus used in this study

The following archaea were used in this study: *Halogranum roseipondis* sp. *nov*. SS5-1^T^ isolated originally in 2011 (5) and reference strains *Haloarcula vallismortis* (21, 22), *Hgn. salarium* B-1 (13, 20), and *Haloferax gibbonsii* LR2-5 (49). *Har. vallismortis* and *Hgn. salarium* were purchased from the Leibniz Institute DSMZ–German Collection of Microorganisms and Cell Cultures GmbH with catalog numbers DSM 3756 and DSM 23171, respectively. HGTV-1 was originally isolated on *Halogranum roseipondis* sp. nov. SS5-1^T^ from a water sample collected from a solar saltern in Samut Sakhon, Thailand (5).

### Growth conditions

MGM was used for cultivating archaeal strains. The base solution for the medium—a 30% (wt/vol) stock solution of artificial seawater (SW) containing 4.5 M salts—was prepared as previously published (5) and diluted to 23% (3.4 M salts), 20% (3.0 M salts), and 18% (2.7 M salts) (vol/vol) for liquid culture and solid and top-layer media, respectively. Solid and top-layer media were obtained by adding 14 or 4 g of Bacto agar (Difco Laboratories) per liter, respectively, and included 5 g peptone (Oxoid) and 1 g Bacto yeast extract (Difco Laboratories) per liter. For assessing growth at different salt conditions, the dilution of SW in the media was adjusted to obtain the desired final salt concentration. Archaeal strains were streaked on MGM-agar plates and grown at 37°C until colonies appeared. The liquid starter cultures were inoculated in 30 mL of MGM broth and grown for 72 h at 37°C with 200 rpm aeration. For growth temperature characterization, the starter culture was grown at the corresponding temperature.

The minimum NaCl requirement to maintain cells intact was tested by harvesting cells from 0.5 mL volume of a starter culture by centrifugation (3,000 × *g,* 5 min, RT) and gently resuspending the cells to NaCl in ddH_2_O (0%–18% NaCl range). Cells were washed by pelleting and gently resuspending them to the desired NaCl concentration. Cell integrity was observed after 30 min and 15 h of incubation at RT using phase-contrast microscopy with Leica DM750 microscope.

Optimal culture conditions were determined using a Bioscreen FP-1100-C photometric microplate absorbance reader by assessing growth (defined as change in OD_600_) at various temperatures, pH, and salinity ranges. Briefly, starter cultures were diluted to an OD_600_ of 0.1–0.2 using fresh medium (corresponding to the condition tested) and loaded onto Bioscreen honeycomb well plates. Alternatively, starter cultures were not diluted but used as such if the OD_600_ was <0.1. All growth curve characterizations were performed with a total culture volume of 380 µL/well. The Bioscreen was set to continuous medium aeration, and amplitude with temperature control was applied according to the condition tested.

### Genome isolation and analysis

The gDNA was isolated by spooling as previously described (50) with minor modifications. Briefly, the 50 mL of exponential culture (OD_600_ = 0.6) was pelleted by centrifugation at 8,000 × *g* for 10 min at 15°C. The cell pellet was resuspended in 2 mL ST buffer (1 M NaCl, 20 mM Tris-HCl, pH 7.5). Cell suspension was lysed by the addition of 2 mL of lysis solution (100 mM EDTA, pH 8.0, 0.2% SDS). The mixture was overlaid with 4 mL of 99.6% ethanol, and gDNA was spooled from the interphase by capillary. The spooled gDNA was washed twice by swirling the capillary in 1 mL of 99.6% ethanol. The gDNA was then dried, resuspended in 1 mL TE buffer, and finally precipitated by the addition of 0.1 vol of 3 M sodium acetate, pH 5.5, and 0.8 vol of isopropanol. After pelleting (6,000 × *g*, 5 min, RT), the gDNA was washed by 1 mL of 70% ethanol and pelleted again. The air-dried pellet was resuspended in 1 mL TE and treated with 1 µL (30 mg/mL) DNase-free RNase A (Applichem) for 1 h at 45°C. The gDNA was reisolated by isopropanol precipitation as described above, and the final pellet was resuspended in TE.

The genome was sequenced using Pacific Biosciences PacBio Sequel II sequencing technology, and assembly was performed using the PacBio Microbial Genome Analysis application integrated in SMRTlink version 11 at the DNA Sequencing and Genomics Laboratory, Helsinki Institute of Life Science, University of Helsinki. The assembled genome was annotated using the prokaryotic genome annotation tool Prokka (51), Galaxy version 1.14.6 + galaxy1. DNA molecules were visualized using DNAplotter (52). This Whole Genome Shotgun project has been deposited at GenBank under the accession number JBUEED000000000.

The phylogeny of *Hgn. roseipondis* was first investigated by pangenome analysis using Roary (30) and further assessed by comparing 16S rRNA sequences of all *Halogranum* species and the reference strains using MUltiple Sequence Comparison by Log-Expectation (MUSCLE) with default settings (53) and by conducting OrthoANI (54). Roary and OrthoANI analyses were applied on a set of 125 type species representing all genera within family *Haloferacaceae* except *Candidatus Haloaextosymbiotes* (see Table S1 at https://doi.org/10.5281/zenodo.21291954).

HGTV-1 genome was retrieved from NCBI GenBank with accession number NC_021328. tRNAscan-SE 2.0 (36) was used in default mode with sequence sources for archaea or mixed settings selected to find tRNA genes in *Hgn. roseipondis* and HGTV-1 genomes, respectively. Codon usage was analyzed with the Codon Usage (Cusp) application from the European Molecular Biology Open Software Suite (55). Sequence comparison of host and virus tRNAs was conducted using MUSCLE (53) with default settings.

### Phenotypic assays

For subsequent assays, *Hgn. roseinpondis* grown on an MGM plate over three to four nights was used. Colonies were streaked on a glass slide and supplemented with a droplet of ~40 µL of 3% H_2_O_2_. Catalase activity was indicated by the release of oxygen, observed as bubbling within the culture mass. The oxidase test was performed by applying 1% N,N,N′,N′-tetramethyl-p-phenylenediamine dihydrochloride (in ddH_2_O) on a cotton swab with archaeal mass from the plate, and the oxidase activity was detected by the formation of a dark purple color.

Next, single-carbon source utilization was determined as described in the Halohandbook (50, 56). Briefly, minimal media including 1 mL/L of trace element solution (2.5 mM MnCl_2_, 2.2 mM ZnSO_4_, 0.2 mM CuSO_4_, and 8.3 mM FeSO_4_) were prepared and supplemented with glycerol (0.3% wt/vol), sodium acetate (0.1% wt/vol), sodium succinate (0.5% wt/vol), galactose (0.4% wt/vol), or 0.3% wt/vol fructose, glucose, lactose, mannitol, or sucrose. Three separate colonies were streaked on a minimal media plate and growth was assessed after a 14-day incubation at 37°C.

Inhibition of the growth by different antibiotics was tested by growing the archaea in 23% MGM liquid broth supplemented with ampicillin 100 µg/mL, streptomycin 50 µg/mL, tetracycline 10 µg/mL, chloramphenicol 35 µg/mL, rifampicin 10 µg/mL, novobiocin 2.5 µg/mL, or mevinolin 4 µg/mL.

Anaerobic growth was assessed by streaking MGM plates and incubating them in an anaerobic jar, from which oxygen was depleted using Microbiology Anaerocult A (Merck) strips according to the manufacturer’s instructions. Potential growth was assessed following a 7-day incubation period.

For starch hydrolysis, archaeal strains were streaked onto 20% MGM agar plates supplemented with soluble starch (2 g/L) and incubated for 1 week at 37°C. Hydrolysis was detected by flooding the plates with Lugol’s iodine solution (57). Gelatin hydrolysis tests were performed by utilizing 20% MGM agar plates supplemented with 0.8% (wt/vol) gelatin, according to the protocol by Dela Cruz and Torres (58). After a 14-day incubation at 37°C, results were visualized by flooding the plate with saturated ammonium sulfate solution.

Motility was assessed as spread of growth on top-layer soft-agar plates by either stabbing colonies into the top-layer agar or by pipetting 10 µL droplets of the early exponential-phase liquid culture on the plates and by observing the cells using light microscopy. Plates were incubated at 37°C for five nights. For light microscopy, samples from plate and liquid culture were prepared. Colonies were mixed in 20% SW, and liquid culture grown to an OD_600_ of 0.6 was directly pipetted on microscopy slide.

### Electron microscopy

TEM and scanning electron microscopy (SEM) of *Hgn. roseipondis* cells were conducted using liquid cultures grown to an OD_600_ of 0.6 (early) or 1.0 (mid-logarithmic), respectively. Cells (5 mL) were collected by centrifugation at 3,400 × *g* for 5 min, resuspended in 23% SW, and fixed in 2.5% glutaraldehyde at 4°C for 18 h. Glutaraldehyde was removed by centrifugation as above and replaced with water. For TEM, 2 µL of the fixed cell sample was pipetted on a carbon-coated, glow-discharged copper mesh grid for 2 min, after which the sample was negatively stained using 3% uranyl acetate for 10 s and rinsed with ddH_2_O. For SEM, fixed cells were immobilized on concanavalin A-coated glass slips, osmicated with 1% OsO_4_ (in water), and dehydrated through an ethanol dehydration series with 50% (vol/vol), 70%, 96%, and 99.6% ethanol.

Virus samples for TEM were produced by purifying the virus from a liquid culture infected at an OD_600_ of 0.6, as has been described before (5). In short, viruses were precipitated from the lysate using polyethylene glycol 6000 (10% wt/vol) and further purified through rate-zonal ultracentrifugation in a linear 5%–20% sucrose gradient prepared in 18% SW (104,000 × *g*, 30 min, 20°C). Light scattering virus zone from the gradient was collected, and viruses were concentrated using differential ultracentrifugation (128,000 × *g*, 75 min, 15°C). The purified virus was resuspended in 18% SW, pipetted on a glow-discharged, carbon-coated Cu-grid, and stained with neutral 2% uranyl acetate.

TEM was performed with JEOL JEM-1400 (Jeol Ltd., Tokyo, Japan) equipped with Gatan Orius SC 1000B CCD camera (Gatan Inc., USA) and SEM with a FEI Quanta 250 Field Emission Gun scanning electron microscope equipped with Gatan 3View system. Electron microscopy was carried out in the Electron Microscopy Unit of the Institute of Biotechnology, University of Helsinki.

### HGTV-1 infection analysis

HGTV-1 plate lysate was prepared by plating suitably diluted virus, yielding semiconfluent plates mixed with 200 µL of host starter culture and 3 mL of 18% MGM top agar (temperature adjusted to 55°C) on 20% MGM agar plates, which were incubated at 37°C for four nights. The top-agar layer from each semiconfluent plate was collected into a flask, to which 1.8 mL of 23% MGM broth/plate was added. Next, viruses were separated from agar and cell debris by shaking the mixture at RT for 2 h at 200 rpm, followed by centrifugation at 8,800 × *g* for 30 min at RT.

To estimate the number of infective viruses, plaque assays were performed for various samples throughout this study. Briefly, a dilution series of the virus sample was made in 23% MGM to yield a countable number of plaques on the host lawn and plated as described above. The average number of plaques produced from each dilution is then applied to count the number of infective viruses in the original sample.

The cross-infectivity of HGTV-1 was also tested on the reference strains (see the section Archaeal Strains and Virus Used in This Study) by conducting a spot-on-lawn assay. The archaeal starter culture was plated with top agar as described above and left to solidify for ~30 min at RT. Different dilutions of HGTV-1 plate lysate were pipetted on each plate as 10 µL droplets, and the infectivity was observed as plaque formation after incubation of the plates at 37°C for five nights.

HGTV-1 infection on *Hgn. roseipondis* was investigated in different salinities and temperatures. *Hgn. roseipondis* liquid cultures were grown to an OD_600_ of 0.6 in growth media of different salinities and temperatures and infected with the plate lysate using a multiplicity of infection of 10. Cultures were then divided on honeycomb plates, and the optical density of the cultures was monitored using Bioscreen FP-1100-C as described earlier.

The length of the intracellular phase of the infection cycle and the burst size of HGTV-1 were determined by conducting a one-step growth curve assay (59), in which the applied adsorption time with light shaking after adding the viruses to the culture was 3 h.

## Data Availability

The genome sequence data and their annotation have been deposited at GenBank under the accession number JBUEED000000000. These data have also been deposited at the open science repository Zenodo (https://doi.org/10.5281/zenodo.20541944).
